# Zinc utilization by microglia in Alzheimer’s disease

**DOI:** 10.1016/j.jbc.2024.107306

**Published:** 2024-04-20

**Authors:** Daniel C. Shippy, Sophia F. Oliai, Tyler K. Ulland

**Affiliations:** 1 Department of Pathology and Laboratory Medicine, School of Medicine and Public Health, University of Wisconsin, Madison, Wisconsin, USA; 2 Wisconsin Alzheimer’s Disease Research Center, School of Medicine and Public Health, University of Wisconsin, Madison, Wisconsin, USA

**Keywords:** Alzheimer’s disease, microglia, neurodegeneration, neuroinflammation, zinc

## Abstract

Alzheimer’s disease (AD) is the most common form of dementia defined by two key pathological characteristics in the brain, amyloid-β (Aβ) plaques and neurofibrillary tangles (NFTs) composed of hyperphosphorylated tau. Microglia, the primary innate immune cells of the central nervous system (CNS), provide neuroprotection through Aβ and tau clearance but may also be neurotoxic by promoting neuroinflammation to exacerbate Aβ and tau pathogenesis in AD. Recent studies have demonstrated the importance of microglial utilization of nutrients and trace metals in controlling their activation and effector functions. Trace metals, such as zinc, have essential roles in brain health and immunity, and zinc dyshomeostasis has been implicated in AD pathogenesis. As a result of these advances, the mechanisms by which zinc homeostasis influences microglial-mediated neuroinflammation in AD is a topic of continuing interest since new strategies to treat AD are needed. Here, we review the roles of zinc in AD, including zinc activation of microglia, the associated neuroinflammatory response, and the application of these findings in new therapeutic strategies.

Alzheimer’s disease (AD) is a degenerative brain disorder and the most common type of dementia. In the United States, approximately 6.7 million people are currently living with AD, and this number is estimated to grow to 13.8 million people by 2060 (1). AD is the seventh leading cause of death in the United States and reported deaths from AD have increased by 145% between 2000 and 2019 (1). AD carries a significant economic impact, as total payments in 2023 for health care, long-term care, and hospice for people aged 65 and older with AD in the United States are estimated to be $345 billion (1). Therefore, there is an urgent need to develop therapeutic strategies that prevent or slow the progression of AD.

AD is a progressive disease caused by damage to neurons in the brain, and changes in the brain can begin to occur 20 or more years before symptoms start (2, 3, 4, 5). AD pathogenesis is complex, and the exact mechanism is still not clear. AD pathology is characterized by the aggregation of extracellular amyloid-β (Aβ) plaques followed by the development of intracellular neurofibrillary tangles (NFTs) composed of hyperphosphorylated tau (6). Amyloid pathology begins with altered cleavage of amyloid precursor protein (APP) by β- and γ-secretases to generate Aβ peptides (7). The resulting Aβ peptides are generally 39 to 43 residues in length (8) with the predominant peptide variants being Aβ_40_ and Aβ_42_ (9). Aβ_42_ is more prone to aggregate than Aβ_40_, and Aβ_42_ is the major component of amyloid plaques in the brain even though Aβ_40_ is significantly more abundant (10, 11). The Aβ_42_/Aβ_40_ ratio is widely considered to play a crucial role in AD pathogenesis with higher ratios correlating with higher neurotoxicity (12) and early onset familial AD (13), while lower ratios correlate with decreased Aβ deposition (14).

NFTs, the other pathological hallmark of AD pathology, are composed primarily of hyperphosphorylated, aggregated forms of the microtubule-binding protein, tau (15, 16, 17, 18). Tau normally promotes assembly and maintains microtubule structure (19), however, in an AD brain, hyperphosphorylated tau polymerizes into NFTs and loses the ability to bind to tubulin or promote tubulin assembly into microtubules (20, 21). Several studies have shown that regions of tau accumulation and high NFT density correlate with disease severity and clinical symptoms in AD (22, 23). There appears to be a connection between tau and Aβ AD pathogenesis, due to the strong correlation in Aβ plaque burden and the severity of tau pathology (24). Aβ plaques have been shown to create an ideal environment for the rapid amplification of tau aggregates (25), while tau aggregates have been shown to mediate Aβ pathogenesis in AD (26, 27). The Aβ/tau synergistic relationship may act to cause NFT-mediated neuron loss, memory/behavior deficits, and synaptic dysfunction (26, 28, 29, 30, 31). Altogether, the pathogenic processes of AD promote the activation of glial cells, such as astrocytes and microglia.

## Microglia in AD

Microglia, the primary innate immune cells of the central nervous system (CNS), originate in the yolk sac and migrate to the CNS during embryogenesis (32). Microglia comprise approximately 5 to 10% of total brain cells and are the only true CNS parenchymal macrophages (33). Microglia continually maintain their CNS population by self-renewal, with very little to no contribution from blood cells (34, 35). During CNS development, microglia play pivotal roles in synapse modulation, complement mediated synapse pruning, and neurogenesis (36). In CNS injury, microglia phagocytose dead cells, microbes, protein aggregates, and other particulates that may threaten the health of the CNS (37).

**Figure 1:**
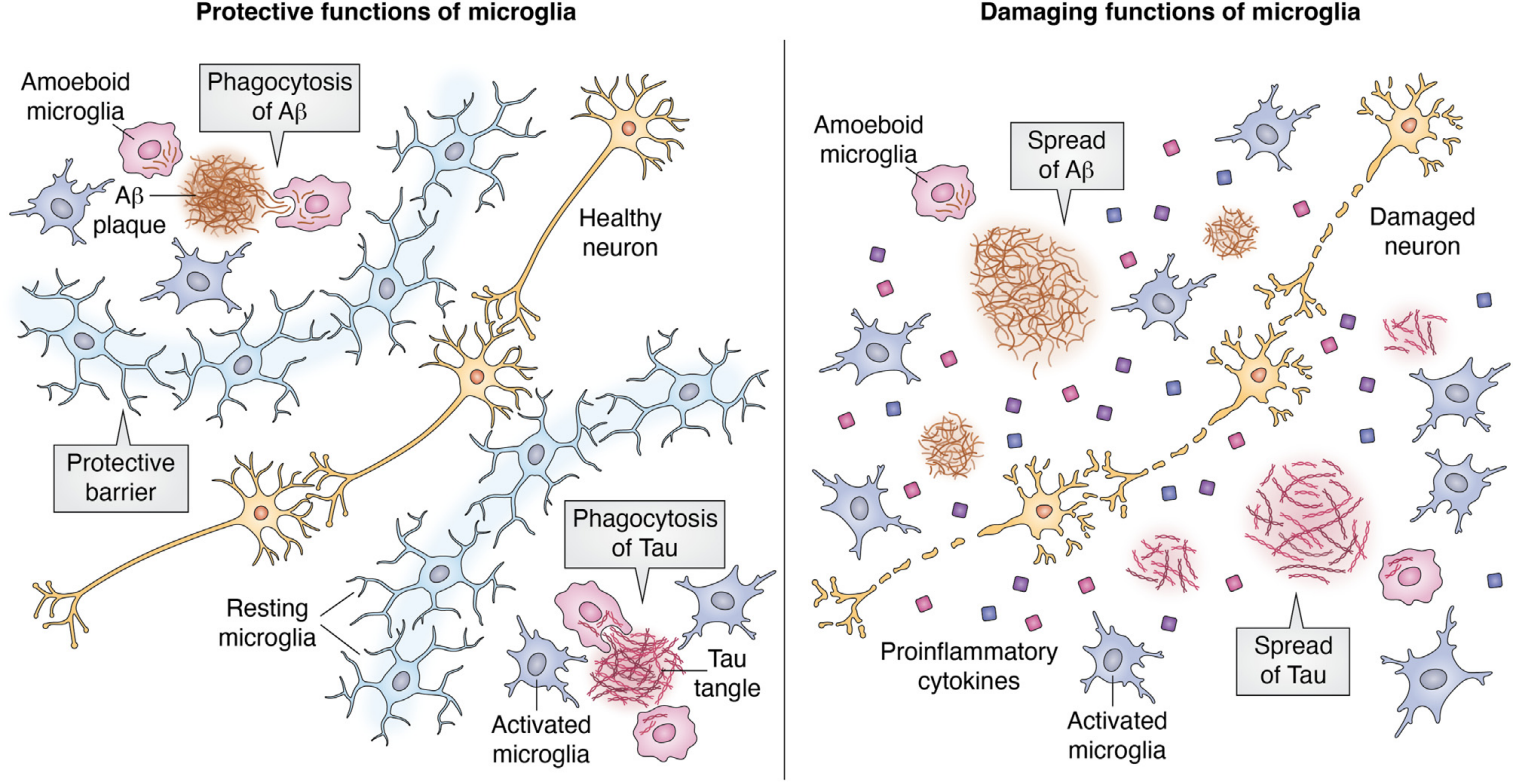
**Dual functions of microglia in AD.** Microglia provide a beneficial response in AD through the phagocytosis of Aβ and tau. Microglia also slow AD progression by forming a physical barrier around plaques to limit their spread and protect surrounding neurons. In contrast, microglia may exacerbate AD progression by facilitating the spread of phagocytized Aβ and tau to unaffected regions of the brain. Microglia also promote neuroinflammation to exacerbate Aβ and tau pathogenesis.

Microglia play a multifaceted role in AD, with the exact mechanisms not entirely understood. Recently, considerable advancements have been made in our understanding of how microglia function and influence AD pathogenesis (38, 39, 40, 41, 42, 43, 44, 45, 46, 47, 48). Alois Alzheimer first described microglial activation in AD over 100 years ago (49). Diverse microglial morphological phenotypes were first described in the human brain in 1919 (50). Microglial morphology is altered during AD progression, suggesting microglia quickly adapt to local environments (50). Based on morphological characteristics, microglia are classified as ramified (resting), activated, and amoeboid (phagocytic) (37). Activated microglia are hypothesized to slow AD progression through the phagocytosis of Aβ and tau (51, 52, 53, 54, 55). Additionally, microglia can form a physical barrier around plaques to limit their neurotoxic effects and protect adjacent neurons while the Aβ is removed from the brain parenchyma (56, 57, 58). There is also evidence that suggests microglia may exacerbate some aspects of AD progression. Studies have shown that phagocytized Aβ and tau are carried around by microglia to unaffected regions of the brain, promoting the spread of plaques (59, 60, 61). Additionally, activated microglia promotes neuroinflammation which contributes to AD progression and severity (62). An overview of the multiple activities of microglia in AD is shown in Figure 1.

Neuroinflammation is defined as an inflammatory immune response within the CNS. Microglia play a central role in neuroinflammation, with the release of proinflammatory cytokines/chemokines, nitric oxide (NO), reactive oxygen species (ROS), and other proinflammatory molecules implicated in AD pathology (63, 64). Several proinflammatory cascades that drive neuroinflammation in AD have been highlighted, such as the nod-like receptor family pyrin domain containing 3 (NLRP3) inflammasome, nuclear factor kappa-light-chain-enhancer of activated B cells (NF-κB), and type 1 interferons (65, 66, 67, 68). Neuroinflammation was initially thought to occur only in the later stages of AD, and glial activation was believed to coincide with, but not significantly contribute to, AD pathology (69, 70). More recent evidence, however, suggests reactive microglia may drive early AD pathogenesis by contributing to neuroinflammation. Reactive microglia are frequently found in large numbers around senile plaques in AD patients, along with elevated levels of proinflammatory cytokines/chemokines (71, 72). Additionally, reactive microglia have been shown to exacerbate amyloid and tau pathology in AD (62, 68, 73). Therefore, microglial neuroinflammatory cascades/pathways are potential therapeutic targets for AD, and numerous studies have shown that reducing neuroinflammation significantly attenuates AD pathology (59, 74, 75, 76, 77).

Translocator protein (TSPO) positron emission tomography (PET) imaging is a powerful tool to determine neuroinflammation by immune cells, such as microglia, in AD (78). TSPO is an 18 kDa protein located primarily on the outer mitochondrial membrane (79). In AD, microglial TSPO expression is upregulated, correlating with the distribution of Aβ and tau (80). TSPO inhibition by reduced exposure to heavy metals, antioxidant supplementation, exercise, and caloric restriction can delay aging and AD progression through the inhibition of apoptosis (81, 82). Originally thought to be a biomarker of microglial activation in humans, recent investigation suggests TSPO PET imaging as an indicator of microglial proliferation (83). Regardless of these findings, TSPO PET imaging in humans is still a valuable tool, as it could be used to determine short-termed responses to anti-inflammatory drugs. Additionally, TSPO PET imaging can be integrated with transcriptomic data and Aβ PET imaging in patients from the same clinical AD cohort to determine relationships between immune cell transcriptomic and brain inflammation changes (84).

Microglial immunometabolism has recently emerged as an important contributor in AD progression and pathogenesis (43, 85, 86, 87). Glucose is the primary energy source for microglia with numerous genes highly expressed for glycolytic and oxidative energy metabolism in the cerebral cortex of the brain (88, 89). Altered patterns in glucose metabolism is a biomarker during the initial stages of AD development (90) with changes in glial metabolism linked to AD pathology and cognitive impairment (91, 92). Several key studies have shown how vital proper microglial metabolism is in AD progression. For example, Ulland *et al.* found that triggering receptor on myeloid cells (TREM2) plays a critical role in microglial metabolic fitness in AD (43). Using 5XFAD mice, a transgenic mouse model of AD characterized by Aβ plaque deposition in the brain (93), their study indicated that microglia from *Trem2*^*−/−*^ 5XFAD mice were less metabolically competent due to significantly reduced mammalian target of rapamycin (mTOR) and glycolytic activity (43). Furthermore, the microglial metabolic deficiency observed in *Trem2*^*−/−*^ 5XFAD mice was restored by increasing microglial energy capacity with cyclocreatine (43). In another study, microglial-mediated acute inflammation and phagocytosis due to Aβ exposure caused an mTOR-dependent metabolic shift from oxidative phosphorylation to glycolysis (87). Once activated, the microglia entered a tolerant state following chronic exposure to Aβ characterized by diminished inflammatory and phagocytic responses. Boosting glycolytic metabolism with interferon-γ, however, restored the phagocytic ability of tolerant microglia (87). These studies, and many others, highlight the importance of microglial immunometabolism in AD and suggest metabolic reprogramming of microglia as a promising therapeutic strategy.

## Zinc

Zinc is a trace metal ion required for all living organisms. Zinc is necessary for numerous biological functions including gene transcription, immunity, and metabolism (94). Dietary intake of zinc is important, as it cannot be stored in significant amounts by the human body. Zinc is found is several foods including fish, legumes, nuts, animal meat, and other dietary sources (95). Compared to other metal ions used by the human body, zinc is generally considered non-toxic, and most effects of chronic zinc toxicity are due to interference with copper uptake, resulting in copper deficiency (96, 97). Zinc toxicity rarely occurs due to the overconsumption of food containing zinc; instead, toxicity occurs with zinc supplementation, and environmental and occupational exposures (98, 99, 100). In contrast, approximately 17% of the global population is at risk for inadequate zinc intake based on zinc content in the national food supply, with citizens of underdeveloped countries most at risk (101, 102). Furthermore, zinc deficiencies are correlated with numerous diseases including obesity (103), diabetes (104), dermatitis (105), cardiovascular disease (106), and neurodegenerative diseases (107).

The brain is the organ with the highest concentration of zinc in the human body, and there is approximately 10-fold more zinc in the brain than in the serum (108, 109). In the human brain, zinc serves as a structural component to approximately 70% of proteins, assisting in the performance of over 300 enzymes and 2000 transcription factors (108, 109). Zinc is essential for synaptic and axonal transmission; tubulin growth and phosphorylation; and nucleic acid metabolism (110). Neurons containing zinc are found in many regions of the brain, including the amygdala, olfactory bulb, and cortex (111). The neurons of the hippocampal mossy fiber pathway, however, house a remarkably high concentration of zinc which modulates the N-methyl-D-aspartate (NMDA) receptor, an important neuronal mechanism responsible for the basis of memory formation (112). Since zinc is crucial for numerous neurobiological processes, zinc homeostasis is tightly regulated by metallothioneins and zinc transporters (109).

Regulation of cellular zinc homeostasis in the brain is carried out *via* two different zinc transporter groups, the zinc transporter (ZnT) family (SLC30A) and the zinc Irt-like protein (ZIP) family (SLC39A). The ZnT family contains 10 proteins (ZnT 1–10) and the ZIP family contains 14 proteins (ZIP 1–14) (113). The ZIP family of zinc transporters moves zinc from the organelles or extracellular space into the cytoplasm, thereby increasing intracellular zinc levels. In contrast, the ZnT family reduces intracellular zinc levels by moving zinc out of the cytoplasm into the extracellular space and organelles (114). Several of these transporters are present in neurons, with ZnT3 necessary for zinc accumulation in synaptic vesicles (115, 116), while ZIP1 and ZIP3 have been shown to control zinc accumulation and toxicity in different subpopulations of neurons in the hippocampus (117). ZIP1 also localizes to the membrane of glial cells to facilitate zinc uptake by astrocytes and microglia (118). Furthermore, ZIP1-mediated uptake of zinc has been shown to activate microglia (discussed further below) *via* the nicotinamide adenine dinucleotide phosphate (NADPH) oxidase and Poly (ADP-ribose) polymerase-1 (PARP-1) pathways (119). Together, these studies, and many others, highlight the importance of zinc uptake and homeostasis in numerous biological processes, including neuron and glial functions.

## Zinc in AD

Synaptic zinc turnover declines with age due to the large energy expenditure required to modulate neurophysiological functions (120). Zinc plays a critical role in chronic diseases related to aging, as intestinal absorption of zinc is decreased with age (121). Serum zinc levels also decline with age, with the decline more pronounced in patients with AD when compared to healthy age-matched controls (122). In contrast, several studies suggest zinc accumulation in the brain as a main feature of AD in post-mortem analyses (123, 124, 125). In the study by Religa *et al.*, AD brains showed a greater than 2-fold increase in cortex tissue zinc levels compared to healthy controls. Their study also showed zinc levels increased with tissue Aβ levels, and higher zinc levels correlated with increased Aβ and overall AD severity (123). Other studies provide more evidence to confirm these findings by showing increased zinc levels in the hippocampus and amygdala of AD brains relative to healthy controls (124, 125). Some studies, however, have reported AD brain tissue has decreased levels of zinc when compared to healthy controls (126, 127). Due to the conflicting data presented in these studies, there is no consensus for zinc content in AD brains. Standard guidelines for sample collection, zinc detection, and zinc measurement will be needed in order to clarify the discrepancies seen in the discussed studies.

In a healthy brain, Aβ is generated from APP in relatively small amounts and is usually quickly cleaved further by Aβ degrading enzymes, most of which are zinc metalloproteases. Common zinc metalloproteases that serve as Aβ degrading enzymes include endothelin-converting enzyme (ECE) 1 and 2, insulin-degrading enzyme (IDE), presequence protease (PreP), neprilysin (NEP), matrix metalloprotease (MMP) 2, three and 9 (128). NEP seems to be the most active Aβ degrading enzyme, with the ability to degrade both monomeric and oligomeric Aβ (129, 130). Aβ plaques contain increased concentrations of zinc, along with other metal ions, such as iron and copper (131, 132). Zinc binds to Aβ (133, 134) and studies show zinc interacts specifically with histidine residues 6, 13, and 14 at the N-terminal of Aβ (135, 136, 137, 138). Once bound, rapid aggregation occurs (139) and intra- and intermolecular zinc coordination in Aβ oligomers decrease the solvation of the oligomer to stimulate further zinc/Aβ aggregation (140). Zinc has also been shown to regulate the degree of self-assembly of Aβ peptides and modulate amyloid morphology *via* distinct coordination sites (141). The role of zinc in Aβ biology appears to be concentration dependent, with zinc contributing to neuroprotection at low (micromolar) and neurotoxicity at high (millimolar) concentrations (142, 143). The precise role of zinc homeostasis in Aβ is not fully understood, but it seems clear that zinc interferes with copper-induced reactive oxygen species (144). Zinc has been shown to compete with copper for Aβ, attenuating oxidation in proximity to plaques by subduing redox activity and peroxide formation (144).

Several studies suggest that dysfunction in zinc homeostasis in the brain results in tau aggregation, which impacts tau levels and promotes the formation of NFTs (145, 146, 147, 148, 149). Zinc activates several kinases including extracellular regulated protein kinase 1/2 (ERK1/2), c-Jun N-terminal kinase (JNK), glycogen synthase kinase-3β (GSK-3β) and other Src-dependent pathways, which are implicated in tau hyperphosphorylation (145, 147, 150, 151, 152, 153). Zinc also indirectly promotes tau hyperphosphorylation through inhibition of major tau phosphatases, such as protein phosphatase 2A (pp2a) (154, 155). Furthermore, zinc is able to directly induce tau aggregation by binding to the repeat, C- and N-terminal regions of tau (156). Recent studies suggest a new mechanism of tau aggregation, where tau condenses into liquid droplets by a process known as liquid-liquid phase separation (LLPS) (157, 158). Additionally, further studies demonstrate zinc promotes LLPS of tau (159, 160, 161), suggesting zinc as an important mediator in tau aggregation. Overall, these mechanisms promote increased levels of hyperphosphorylated tau that may enhance tau-associated pathogenesis in AD.

Ferroptosis is a form of iron-dependent cell death induced by erastin (162). Typical features of ferroptosis include ROS generation, inactivation of GPX4, changes in cellular morphology, iron-dependent accumulation of lipid peroxides, and depletion of glutathione (162, 163). Ferroptosis in AD has been extensively studied, with the typical features of ferroptosis listed above observed in the brains of patients with AD (164, 165). Intracellular Aβ cytotoxicity promotes ferroptosis in neurons (166) and several reports show microglia may be susceptible to ferroptosis-mediated cell death (162, 167, 168). Indeed, microglia have been shown to drive ferroptosis-dependent neurodegeneration, and the vesicle trafficking gene, *SEC24B*, has been identified as a central ferroptosis regulator in microglia (168). Microglia are susceptible to iron dyshomeostasis, and iron accumulation in microglia leads to a metabolic switch towards glycolysis and a proinflammatory phenotype (169, 170). Zinc can greatly influence iron levels in AD, as zinc has been shown to inhibit APP ferroxidase activity (171). Furthermore, zinc can inhibit ferroptosis by activating the nuclear factor erythroid-2-related factor 2/heme oxygenase 1 (Nrf2/HO-1) and GPX4 signaling pathways (172). Since Aβ plaques contain increased concentrations of zinc and iron (131, 132), the interplay between these metal ions in ferroptosis are an interesting avenue of research in AD pathogenesis.

Vascular brain injury (VBI) is associated with AD pathology, dementia, and cognitive decline (173, 174, 175). VBI is an important component of AD pathogenesis, as degradation of the neurovascular unit (NVU) leads to deterioration of nerve endings and neuronal cell death (176, 177). Additionally, AD risk factors (diabetes, hypertension) cause blood-brain barrier (BBB) dysfunction and damage the NVU during aging (177, 178). Several studies suggest excess zinc release following transient ischemia contributes to neuronal cell death (179, 180). The study by Zhao *et al.* demonstrated a novel mechanism of VBI by zinc following cerebral ischemia (181). In their study, zinc accumulation during cerebral ischemia injury induced endoplasmic reticulum (ER) stress resulting in ER stress-associated apoptosis in neurons (181). ER stress is observed in the brains of patients with AD, with significantly lower levels of the ubiquitin ligase HRD1 in the cerebral cortex of patients with AD, resulting in Aβ accumulation (182). Together, these studies highlight the importance of zinc in the interplay between AD and risk factors for AD, such as VBI.

## Zinc-associated neuroinflammation in AD

**Figure 2:**
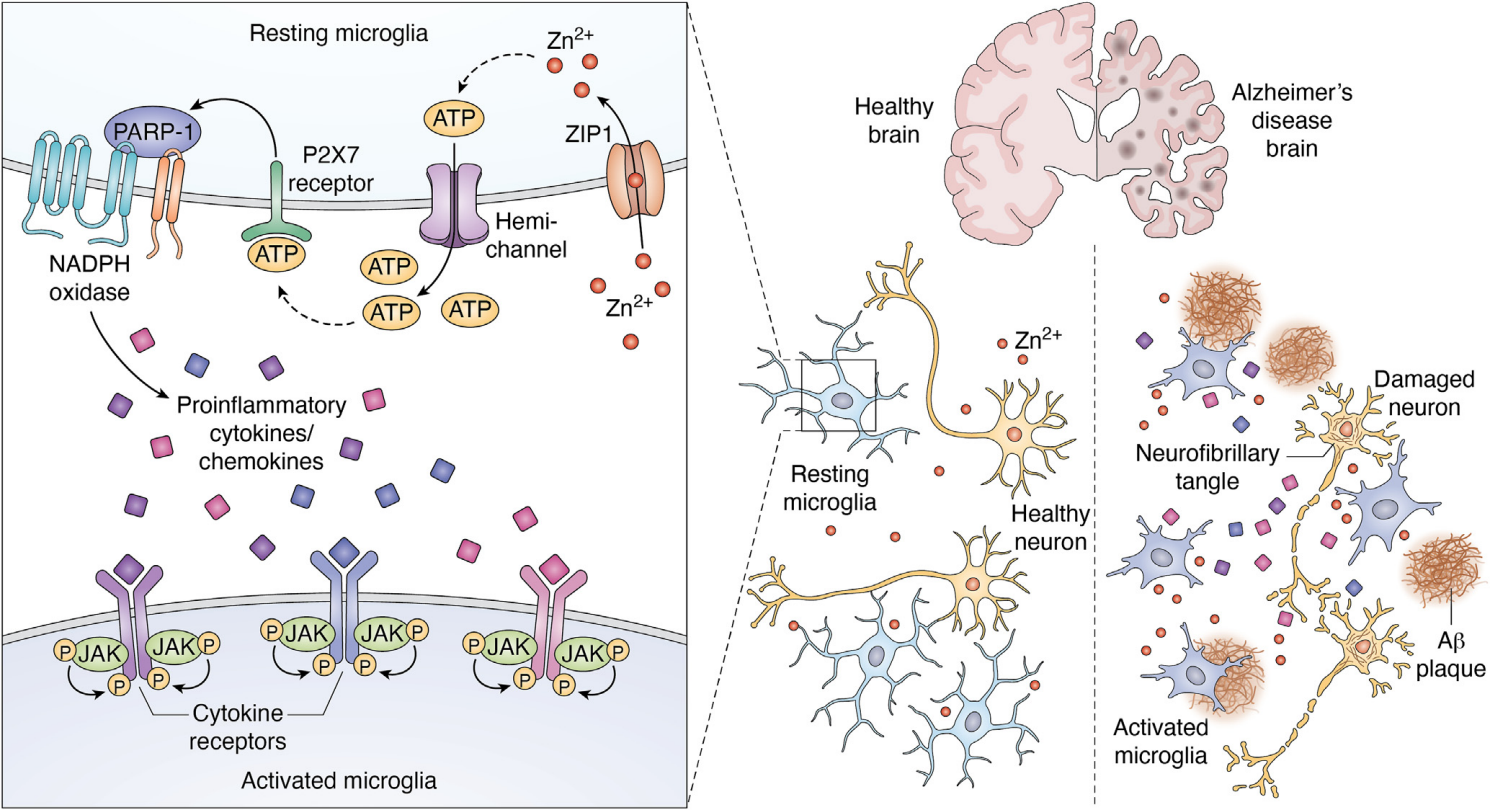
**Zinc activation of microglia.** Extracellular zinc activates resting microglia resulting in neuroinflammation (release of proinflammatory cytokines/chemokines) which exacerbates Aβ and tau pathology in AD. Zinc uptake by microglia is mediated *via* ZIP1 which results in ATP release and subsequent activation of the P2X7 receptor, PARP-1, and NADPH oxidase.

Zinc homeostasis is necessary for proper immune cell functions, with zinc deficiencies responsible for altered cell signaling/activation and enhancement of the proinflammatory response (183, 184). In microglia, zinc has been shown to directly promote microglial activation with increased glycoprotein F4/80 expression, NO production, and NF-κB activity (185). Another study demonstrated zinc chloride treatment enhanced the release of proinflammatory mediators (TNF-α, IL-6, IL-1β) in LPS-stimulated microglia, suggesting zinc primes microglia to release proinflammatory mediators *via* P2X7 receptor activation by modulating ROS generation (186). The study by Higashi *et al.* provides valuable insights into the mechanism of zinc activation of microglia (119). In their study, extracellular zinc was taken up by microglia *via* ZIP1, which in turn stimulated hemichannel-mediated adenosine triphosphate (ATP) release from microglia. This subsequently activated P2X7 receptors in an autocrine/paracrine manner, which activated NADPH oxidase and PARP-1, resulting in microglial activation (119). Activated microglia are a major source of proinflammatory cytokines/chemokines that trigger the progression of several neuroinflammatory cascades which may exacerbate AD pathogenesis (33, 37, 62, 69). An overview of zinc activation of microglia and how it influences AD pathogenesis is shown in Figure 2.

Microglial NF-κB is a major transcription factor that drives the neuroinflammatory response to exacerbate Aβ (187) and tau (68) pathology in AD. As previously discussed, the study by Kauppinen *et al.* suggests zinc promotes microglial activation through the activation of PARP-1, NADPH oxidase, and NF-κB (185). In their study, microglia exposed to zinc (15–30 μM) displayed an activated morphology similar to that induced by LPS (50 ng/ml) (185). Furthermore, inhibiting NF-κB activation with BAY 11-7085 (5 μM), an inhibitor of I-κB phosphorylation, prevented zinc-induced microglial activation. These data suggest a novel trigger for microglial activation and propose a new mechanism by which zinc can contribute to microglial-mediated neuroinflammation in AD (185). Several studies have demonstrated flavonoid compounds, like chrysin, exert anti-inflammatory effects through the attenuation of the NF-κB signaling pathway in microglia (188, 189). In the study by Li *et al.*, the molecular mechanism of NF-κB attenuation by chrysin in microglia was elucidated, suggesting chrysin inhibited NF-κB induction *via* up-regulation of zinc-finger protein A20. Furthermore, their study suggests A20 as a potential target for therapeutic interventions in AD (189). Another study by Hongxia *et al.* also found A20 as an anti-inflammatory factor in LPS-stimulated microglia (190). In their study, zinc supplementation (10 and 30 μM) of LPS-stimulated BV2 microglial cells blocked ROS generation and reduced the secretion of several proinflammatory cytokines (IL-6 and TNF-α). Furthermore, zinc supplementation resulted in increased A20 expression which coincided with negative regulation of NF-κB *via* phosphorylation levels of p65 and IκB (190). Together, these data suggest an important role for zinc in NF-κB activation/regulation in microglial-mediated neuroinflammation.

**Figure 3:**
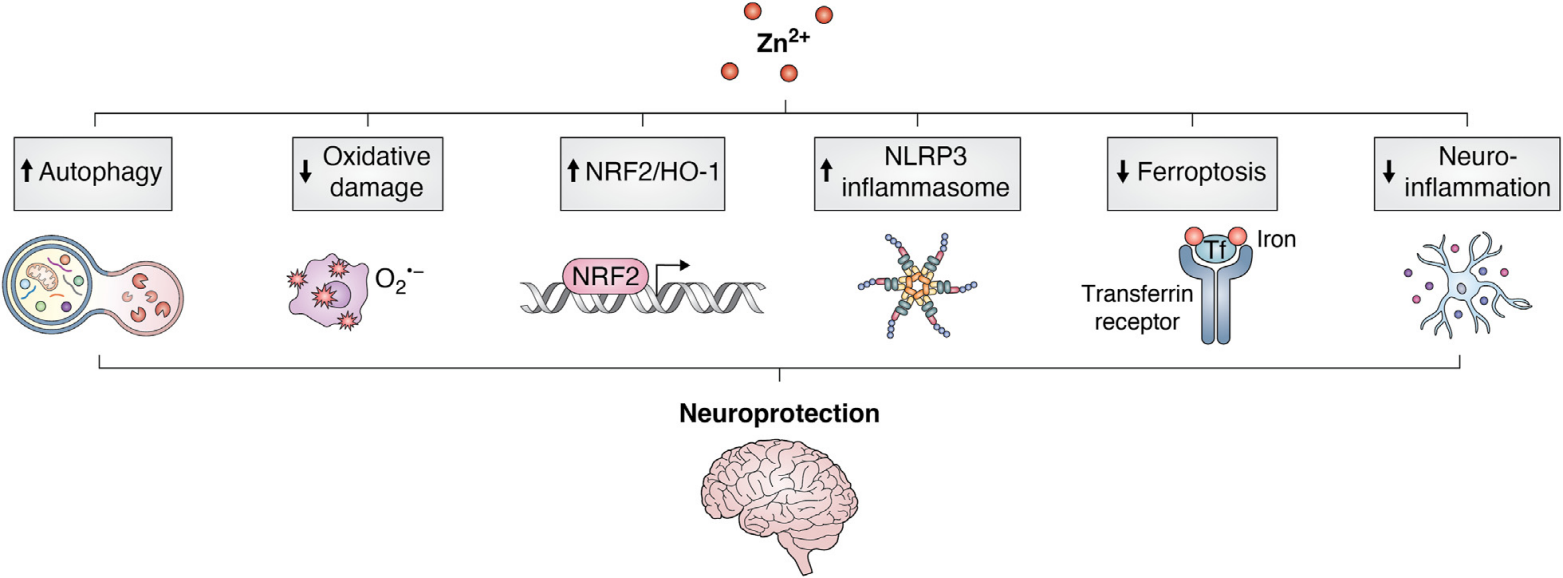
**Potential mechanisms of zinc-mediated neuroprotection.** Zinc is reported to provide neuroprotection to neurons and glial cells by inhibiting ferroptosis and NLRP3 inflammasome activation through autophagy and up-regulation of the Nrf2/HO-1 signaling pathway. Zinc modulation of these mechanisms inhibits oxidative stress and attenuates neuroinflammation.

The NLRP3 inflammasome is activated in microglia in response to damage-associated molecular patterns (DAMPs) and pathogen-associated molecular patterns (PAMPs). Several studies suggest activation of the NLRP3 inflammasome as a major contributor to the neuroinflammatory response which exacerbates AD pathology (65, 74, 191, 192, 193). Initial studies demonstrated zinc metabolism regulates caspase-1 activation and IL-1β secretion (194) and zinc depletion in macrophages activates the NLRP3 inflammasome and promotes IL-1β secretion from cells (195). In a follow-up study by the same research group, Rivers-Auty *et al.* used epidemiological data suggesting zinc supplementation in humans resulted in attenuated symptomatic decline and reduced overall prevalence of AD (196). Their data also demonstrated that zinc deficiency accelerated cognitive decline by potentiation of NLRP3-dependent inflammation in an *APP/PS1* mouse model of AD. Notably, the effect was reversible, as zinc supplementation protected *APP/PS1* mice against cognitive decline by inhibiting NLRP3 inflammasome activation (196). In an effort to understand the mechanism of zinc deficiency in NLRP3 inflammasome activation in *APP/PS1* mice, Rivers-Auty *et al.* performed RNA-sequencing on whole hippocampal homogenates but found no transcriptional changes between genotype and diet (196). Further transcriptional analyses, however, determined significant alterations in several genes important in microglial metabolic fitness and neuroinflammation during AD progression, including *Trem2* (43), *Il1β* (197), and *Nlrp3* (65) from the plaque bearing regions of *APP/PS1* mouse brains (196). Additional mouse experiments showed the memory impairment due to zinc-deficiency in the *APP/PS1* mice was not seen in zinc-deficient *Nlrp3* knockout (*APP/PS1/Nlrp3*^*−/−*^) mice. Finally, *in vitro* experiments showed zinc supplementation inhibited NLRP3 inflammasome activation in bone marrow-derived macrophages (BMDMs) and mixed glial cell experiments (196). Therefore, sufficient evidence exists to suggest zinc activates the NLRP3 inflammasome in AD, but the molecular mechanism is not completely understood. Zinc depletion in macrophages has been shown to disrupt lysosome integrity which is considered a major event in NLRP3 inflammasome activation (195). Further insights into the molecular mechanism could be gained from examining zinc/NLRP3 inflammasome studies using murine spinal injury models, as considerable work has been done in this field (198, 199, 200, 201). In the study by Zhao *et al.*, zinc supplementation of BV2 cells, an immortalized microglial cell line, protected cells from LPS-induced damage by inhibiting apoptosis and promoting autophagy through the down-regulation of *Xist* (198). The down-regulation of *Xist* promoted microglial autophagy-mediated NLRP3 inactivation by regulating miR-374a-5p, suggesting neuroprotection by zinc through inhibition of *Xist*/miR-374a-5p-mediated NLRP3 inflammasome activation (198). Other reports suggest zinc promotion of autophagy as a mechanism of neuroprotection through inhibition of the NLRP3 inflammasome in neurons and glial cells (199, 200) (Fig. 3). Zinc can also provide neuroprotection after spinal cord injury by inhibiting oxidative damage, ferroptosis and NLRP3 inflammasome activation through up-regulation of the Nrf2/HO-1 signaling pathway (172, 201) (Fig. 3). Whether these mechanisms in spinal injury models are similar in AD remains to be investigated, but it appears zinc homeostasis plays a crucial role in NLRP3 inflammasome activity.

## Therapeutic potential of zinc in AD

AD drug development has been especially challenging with most AD drug research focused on Aβ and tau reduction. Aducanumab (Aduhelm) was recently approved for the treatment of AD in the United States, and it is the first treatment approved to target the underlying etiology of AD: Aβ plaques in the brain (202). The FDA’s approval of aducanumab was controversial, as legitimate questions remain regarding the efficacy and safety of aducanumab, leading to its discontinuation in 2024 (203). Two other anti-Aβ antibody therapies, lecanemab and donanemab have recently been approved and appear more efficacious than aducanumab (204, 205, 206, 207). AD is a complex, multifaceted disease, however, and there is still an urgent need for new therapeutic interventions for AD.

Zinc levels have been extensively investigated as a potential biomarker of AD. Studies in serum or plasma have produced inconsistent results, however, as data show significant reductions (208, 209, 210, 211), significant increases (212), or no difference (213, 214) when comparing AD *versus* healthy samples. Furthermore, similar inconsistencies are seen when comparing cerebrospinal fluid (CSF) zinc levels between AD and control samples (215, 216, 217). In an effort to resolve the issue, Rembach *et al.* determined serum zinc concentrations in samples from healthy controls (n = 753), mild cognitive impairment (n = 126), and AD (n = 205) participants in the Australian Imaging, Biomarkers and Lifestyle (AIBL) study (218). The results of their study found reduced serum zinc levels were simply a byproduct of aging since correcting for age-dependent decline resulted in no significant difference in serum zinc levels amongst the three groups (218). Although it appears zinc levels in serum/plasma or CSF may not be reliable biomarkers for AD, these findings do not underscore the impact altered zinc levels in the brain may play in AD pathogenesis.

Transcriptome analyses of microglia have uncovered numerous phenotypic characteristics of microglial responses in the AD brain (38, 39, 43, 44, 87, 219, 220, 221, 222, 223). Further sequencing studies have identified several microglial zinc genes which may be important in AD pathogenesis and could be biomarkers of AD or potential targets for new AD drugs (224, 225, 226, 227). For example, the study by Bottero *et al.* identified zinc-finger transcription factor (YY1) as a transcription factor that potentially regulates key genes in AD pathology (227). Furthermore, their analysis also identified therapeutic agents that target these genes for potential use in AD treatment regimens (227). We previously identified 19 altered zinc-related genes in microglia common to three distinct data sets (cultured cells, mice, humans) comparing an AD *versus* non-AD state. Further bioinformatics analyses identified drugs which target altered zinc-related genes, highlighting potential new AD drug regimens to reduce/eliminate microglial-mediated neuroinflammation in AD associated with zinc utilization pathways (224).

Since zinc dyshomeostasis has been implicated in AD, numerous dietary zinc supplementation studies have been conducted in mice and humans with no clear consensus on the effects of zinc supplementation in AD pathology (122, 228, 229, 230, 231, 232, 233, 234, 235, 236, 237, 238). For example, several studies show dietary zinc supplementation reduced Aβ plaques and/or tau phosphorylation in mouse models of AD (229, 230, 231). In contrast, several studies found dietary zinc supplementation increased AD pathological hallmarks, such as Aβ and NFTs, in mouse models of AD (228, 235, 236), while the study by Maynard *et al.* demonstrated chronic exposure to high levels of dietary zinc had minimal effect on zinc levels and Aβ accumulation in mouse brains (232). In human AD studies, a few small clinical trials using zinc supplementation have reported improvements in cognition and memory following supplementation, but larger clinical trials are needed (122, 237, 238). In contrast to zinc supplementation, zinc chelation by metal protein attenuating compounds (MPACs), such as clioquinol and PBT2, have been also investigated as a therapeutic strategy for AD (228, 239, 240, 241, 242, 243). Clioquinol and PBT2 are prominent metal ionophores (244) that can rapidly chelate zinc and have been shown to improve cognition and reduce Aβ (239, 240, 241, 242, 243). In human clinical AD trials, however, the clioquinol trial was halted due to safety concerns and observing little to no effect between the placebo and treatment groups (245). The PBT2 trial was more thoroughly conducted, and PBT2 appears to be safe and well tolerated in patients with mild AD, but it did not have a dynamic effect on cognition when comparing the placebo and treatment groups (245). Overall, metal chelation appears to work well in animal models of AD and could be a viable treatment strategy (246), but questions still remain as clinical trials in humans have raised efficacy and safety concerns (247).

Recently, a gene regulation therapy using zinc finger protein transcription factors (ZFP-TFs) was described as a new treatment strategy for AD (248). In the study by Wegmann *et al.*, adeno-associated viruses (AAVs) encoding engineered zinc finger protein (ZFP) were used to target the tau gene, *MAPT* (248); mutations in *MAPT* result in tau aggregation and extensive neurodegeneration (249). A single dose of AAV ZFP-TF reduced tau levels in mouse models of AD by 50 to 80%, and neuronal damage around Aβ plaques was restored. Furthermore, the therapy appears to be safe, as no neuroinflammation, neurotoxicity, histopathological changes, or molecular alterations were observed (248). Although these findings represent an exciting new approach to treat AD and other tauopathies, studies to date have only been conducted in mice, and have numerous barriers to cross prior to human use.

Numerous barriers remain in the development of new AD therapies. Many promising AD therapies developed in rodent models do not translate to humans (250). Although transgenic rodent models have been a valuable tool in AD research, AD is a complex disease, and none of the available mouse models truly epitomize the full spectrum of AD pathogenesis (neuroinflammation, Aβ deposition, synapse loss, NFT formation, tau phosphorylation) (250, 251). Human clinical trials for AD are more expensive, slower to enroll study participates, and take longer to complete than in most other therapeutic areas (252). In regards to zinc AD therapies, several other hurdles must be cleared on top of the barriers described above. Most studies conclude zinc dyshomeostasis influences AD pathology, yet there is no consensus on the mechanistic effects of zinc in AD (253). For example, most studies conclude cerebral zinc concentrations change in AD patients, but other studies found no change in the frontal cortex, cortex, or CSF Ventricular fluid (253). Defined guidelines of zinc detection, sample collection, patient demographics (age, sex), and cognitive testing are needed in order to standardize measurements of outcomes for zinc therapies of AD (253).

## Conclusion

Metal homeostasis in the CNS is a crucial component of healthy brain function. Dysregulation of essential metal ions, such as zinc, disrupts neural networks and promotes pathological events, potentially leading to neurodegeneration. Microglia, the primary immune cells in the CNS, play a crucial role in AD pathogenesis and may have beneficial and/or detrimental effects during AD progression dependent on a variety of factors, including activation and metabolic states. Thus, investigation of microglial utilization of zinc during AD progression and pathogenesis is of great interest. As discussed above, zinc activates microglia to promote neuroinflammation, and zinc deficiency accelerates cognitive decline by activation of the NLRP3 inflammasome. Furthermore, interventions that alter zinc concentrations or target important zinc genes, and gene therapy strategies using engineered ZFP may lead to alternative treatment strategies for AD. Since zinc homeostasis in the brain is tightly regulated, and zinc is involved in all aspects of AD pathogenesis (Aβ, tau/NFTs, neuroinflammation), more research is needed to elucidate the mechanisms underlying the multifunctional roles of zinc in AD.

## Data availability

All representative data are contained within the article.

## Conflict of interest

The authors declare that they have no conflicts of interest with the contents of this article.
